# Association of Adiposity with Pulse Pressure Amongst Gujarati Indian Adolescents

**DOI:** 10.4103/0970-0218.69267

**Published:** 2010-07

**Authors:** Wasim A Shaikh, Minal Patel, SK Singh

**Affiliations:** Department of Physiology, Pramukhswami Medical College, Karamsad - 388 325, Gujarat, India

**Keywords:** Adiposity, pulse pressure, vascular distensibility, gender, Gujarati Indian adolescents

## Abstract

**Background and Aim:**

The current study was conducted to determine the effect of adiposity on vascular distensibility in Gujarati Indian adolescents as research indicating the pathogenesis of hypertension among overweight and/or obese Indian adolescents is scant and ethnic differences exist in the pathogenesis of hypertension

**Materials and Methods:**

A cross-sectional study was conducted on 488 Gujarati Indian adolescents of 16-19 years age group. Adiposity was assessed in terms of BMI, Body Fat %, Fat Mass, Fat Mass Index and Waist Circumference. Arterial blood pressure was recorded and pulse pressure (PP) was calculated using the standard equation based on the difference between systolic blood pressure (SBP) and diastolic blood pressure (DBP). Pearson’s correlation coefficient was determined to find the association between the markers of adiposity and SBP, DBP and PP.

**Result:**

A significant positive correlationship was found between adiposity and PP in boys. However, no significant correlationship was found between adiposity and PP in girls.

**Conclusion:**

An increase in total as well as visceral adiposity is probably associated with a decrease in vascular distensibility in the Gujarati Indian adolescent boys but not in girls, thus indicating a protective role of female sex hormone estrogen which has been shown earlier to protect the vasculature from atherosclerosis, endothelial dysfunction which occurs with increase in adiposity.

## Introduction

Studies from various communities across India indicate an increase in prevalence of hypertension among the children and adolescents.(1) An increase in body weight and sedentary lifestyle show strong associations with hypertension among the children and adolescents.(2–4)

Several patho-physiological changes like increase in sympathetic activity, decrease in arterial elasticity and hyperinsulinaemia associated with increase in body mass and unhealthy lifestyle have been implicated in the pathogenesis of hypertension.(5) However, research studies have also reported the existence of differences in the etiopathogenesis of disease across varied ethnic populations and age groups.(6,7) Since scant studies have reported regarding the pathogenesis of adiposity associated rise in blood pressure among Indian adolescents, it is essential to learn the mechanism through which increase in adiposity may be effecting the blood pressure among the Indian adolescents so as to help in the management of overweight and/or obesity associated rise in blood pressure among Indian adolescents.

We studied the association of adiposity with pulse pressure, a reliable indicator of vascular distensibility(8) with the objective to determine if increase in adiposity affects vascular distensibilty among Gujarati Indian adolescents at an age as early as late adolescence.

## Materials and Methods

A cross-sectional study was conducted on 488 Gujarati Indian adolescents (Boys=296 and Girls =192) of 16 to 19 years age group, studying at the schools and colleges in the local population after the approval of institution’s human research ethics committee and obtaining informed consent from the participants or the guardian.

Adiposity was assessed in terms of Body Mass Index (BMI), Body Fat % (BF %), Fat Mass (FM), Fat Mass Index (FMI) and Waist Circumference (WC). BMI was calculated as the weight (kg) divided by the square of height in meters (m^2^). WC was measured at the midpoint between the lower costal margin and the iliac crest to the nearest 0.5 cm at the end of normal expiration. BF % and FM were assessed by bioelectrical impedance technique using Omron Body Fat Monitor HBF -302. FMI was calculated as the fat mass (kg) divided by the square of height in meters (m^2^).(9)

Systolic blood pressure (SBP) and diastolic blood pressure (DBP) were recorded at the brachial artery in the left arm using the Omron T8 with Intellisense (HEM757A4-C1) Automatic Blood Pressure Monitor (Accuracy, BP: ± 4mmHg) according to the recommended guidelines. Blood pressure was recorded at intervals of 1 minute till the difference between two consecutive BP readings was less than 5 mm Hg. The average of the two consecutive readings was used for statistical analysis. Pulse pressure (PP) was obtained by calculating the difference between the average systolic blood pressure and average diastolic blood pressure.(10–12)

Pearson’s correlation coefficient was used to determine the correlationship of markers of adiposity with SBP, DBP and PP.

## Results

A significant correlationship was found between adiposity and SBP, DBP and PP among boys while adiposity showed a significant correlationship with SBP and DBP in girls, but not with PP as shown in Table 1.

**Table 1:** Correlationship of adiposity markers with pulse pressure among Gujarati Indian adolescents

Study variable	Boys (*N*=296)			Girls (*N*=192)		
	SBP	DBP	PP	SBP	DBP	PP
Weight	0.24***	0.09	0.25***	0.28***	0.35***	−0.03
BMI	0.31***	0.14*	0.27***	0.32***	0.38***	−0.01
BF%	0.26***	0.11	0.2**	0.27***	0.35***	−0.05
FM	0.28***	0.11	0.26***	0.3***	0.37***	−0.03
FMI	0.28***	0.12*	0.24***	0.3***	0.37***	−0.03
WC	0.2**	0.08	0.18**	0.2**	0.22**	0.00003

Values indicate Pearson’s correlation coefficient, *r*;

*** *P*<0.001

** *P*<0.005

* *P*<0.01

## Discussion

The present study indicates that adiposity decreases vascular distensibility among the Gujarati adolescent boys at an age as early as 16-19 years. These results are consistent with earlier reports that relate adiposity to vascular distensibility in adolescents. Whincup *et al*.(13) found a strong inverse relationship of BF%, BMI, WC and sum of four skinfolds with the arterial distensibilty in 471 British children in the age group of 13-15 years. Zebekakis *et al*.(14) investigated whether the relationship between arterial stiffness and body mass index (BMI) was consistent across an age range from 10 to 86 years.

The study showed that arterial diameter increased with BMI in all territories, with an opposite trend for arterial distensibility. In men and women, the relationships of brachial and femoral properties with BMI were consistent across the whole age range. In men and women, carotid distensibility decreased more with BMI at young than old age. In middle-aged and older women, but not in men of any age, pulse wave velocity increased with higher BMI. Urbina *et al*.(15) conducted a study on 969 black and white subjects of 13-22 years to identify the association of brachial artery distensibility with atherosclerotic risk factors. The study showed that decreased brachial artery distensibility correlated with male gender, overweight, high blood pressure, heart rate (HR), fasting glucose and log of fasting insulin. Regression modeling found PP and HR to be the major determinants of brachial distensibility.

The probable reason for this gender difference observed in the relationship of adiposity with PP is the fact that the female sex hormone estrogen is believed to protect the post-pubertal female vasculature from atherosclerosis and make the blood vessels more distensible while the male sex hormone testosterone predisposes the male vasculature to endothelial dysfunction and atherosclerosis. Ahimastos *et al*.(16) reported the findings of their study which was conducted to determine the gender differences in large artery stiffness in pre and post-puberty stages. The study showed that pre-pubertal males and females did not differ in body size, cardiac output, or heart rate. It also showed that the pre-pubertal females had stiffer large arteries and higher pulse pressure than age-matched males. However, on the other side, post-pubertal males were taller and heavier and had a greater cardiac output and lower heart rate compared with similarly aged females. In relation to pubertal status, females developed more distensible large arteries post- puberty whereas males developed stiffer large vessels.

Herman *et al*.(17) have demonstrated that the dilatation of brachial artery in response to increase in blood flow (endothelial dependent dilatation) was higher among individuals who were having low levels of serum testosterone. This findings suggested that testosterone causes endothelial dysfunction.

However, though we have not studied the stroke volume in our study population and since stroke volume is the other determinant of pulse pressure apart from vascular distensibilty, the finding that adiposity has strong and equal correlationship with SBP in both boys and girls probably reflects the fact that adiposity associated rise in stroke volume due to volume expansion may not be the reason for the presence of a strong correlationship between adiposity and PP in boys and absence of such relationship in girls.

## Conclusion

This study shows that adiposity affects vascular distensibility in Gujarati Indian adolescents and that the male vasculature is prone to the adverse effects of adiposity in comparison to female vasculature at an age as early as adolescence.

### Limitations and future perspectives

We have indirectly assessed the vascular distensibilty in this study and therefore a much detailed study is required in this population to understand the finer details regarding the effect of adiposity on vascular distensibility.
